# Long non-coding RNA PVT1 interacts with MYC and its downstream molecules to synergistically promote tumorigenesis

**DOI:** 10.1007/s00018-019-03222-1

**Published:** 2019-07-15

**Authors:** Ke Jin, Shufei Wang, Yazhuo Zhang, Mengfang Xia, Yongzhen Mo, Xiaoling Li, Guiyuan Li, Zhaoyang Zeng, Wei Xiong, Yi He

**Affiliations:** 1 NHC Key Laboratory of Carcinogenesis (Central South University) and Hunan Key Laboratory of Translational Radiation Oncology, Hunan Cancer Hospital and the Affiliated Cancer Hospital of Xiangya School of Medicine, Central South University, Changsha, Hunan China; 2 The Key Laboratory of Carcinogenesis and Cancer Invasion of the Chinese Ministry of Education, Cancer Research Institute, Central South University, Changsha, Hunan China; 3 Hunan Key Laboratory of Nonresolving Inflammation and Cancer, Disease Genome Research Center, The Third Xiangya Hospital, Central South University, Changsha, Hunan China

**Keywords:** PVT1, MYC, Gene fusion, Positive feedback, Promoter, Enhancer

## Abstract

Numerous studies have shown that non-coding RNAs play crucial roles in the development and progression of various tumor cells. Plasmacytoma variant translocation 1 (*PVT1*) mainly encodes a long non-coding RNA (lncRNA) and is located on chromosome 8q24.21, which constitutes a fragile site for genetic aberrations. *PVT1* is well-known for its interaction with its neighbor *MYC*, which is a qualified oncogene that plays a vital role in tumorigenesis. In the past several decades, increasing attention has been paid to the interaction mechanism between PVT1 and MYC, which will benefit the clinical treatment and prognosis of patients. In this review, we summarize the coamplification of *PVT1* and *MYC* in cancer, the positive feedback mechanism, and the latest promoter competition mechanism of *PVT1* and *MYC*, as well as how PVT1 participates in the downstream signaling pathway of c-Myc by regulating key molecules. We also briefly describe the treatment prospects and research directions of PVT1 and MYC.

## Introduction

In the entire human genome, only 2% of the genes are used to encode proteins, and the vast majority of the human genome is transcribed into non-coding RNAs, such as long non-coding RNAs (lncRNAs), which are longer than 200 nucleotides (nt) [1]. These lncRNAs play an important role in regulating cellular activities by modulating gene expression, including cell differentiation, proliferation, cell cycle, apoptosis, migration, and invasion [2, 3]. However, abnormal expression of lncRNAs can contribute to the occurrence and development of various diseases, such as cancer [4–6].

The plasmacytoma variant translocation 1 (*PVT1*) gene encodes a lncRNA and was first discovered as an activator of *MYC* in murine plasmacytoma variant translocation in 1984 [7]. The human *PVT1* is a large locus more than 30 kb in length and is located at 8q24.21 [8]. The locus constitutes a fragile site for genetic aberrations, including translocation, amplification, viral integration, and multiple risk loci in cancer or other diseases [8, 9].

Studies have shown that PVT1 is expressed at low levels in normal tissues, while it is highly expressed in various malignant tumors and tumor cell lines, such as gastric cancer, lung cancer, hepatocellular carcinoma, thyroid carcinoma, breast cancer, and pancreatic cancer [7, 9–16]. PVT1 can also serve as a potential predictor of cancer progression and patient prognosis [10, 14, 15]. Most studies have reported that PVT1 can promote proliferation, angiogenesis, apoptosis escape, and participate in DNA rearrangements, which might ultimately promote carcinogenesis [7, 10, 11, 13, 17, 18]. However, recent studies have shown that *PVT1*, also a microRNA Host gene, can encode miR-1204, miR-1205, miR-1206, miR-1207-5p, miR-1207-3p, and miR-1208 [7, 19–21]. Moreover, *PVT1* can also engender a circRNA called circPVT1 by circularizing exon 3 along with long introns on each side [22]. CircPVT1 can act as a miRNA sponge to regulate gene expression and promote cell proliferation in cancer [22, 23].

In the human genome, *MYC* is located only 53 kb upstream of *PVT1*. Both genes have been reported to play a role in cancer [1]. High expression of PVT1 can increase c-Myc expression by regulating c-Myc stability, and they can also interact with each other to regulate their expression, which synergistically promotes the occurrence and development of tumors [7, 24]. *MYC*, a proto-oncogene, was first discovered as the cellular homolog of the Avian virus myelocytomatosis oncogene and plays various roles in protein synthesis, metabolism, and cellular differentiation [25, 26]. C-Myc, a transcription factor, is thought to regulate the expression of 15% of all genes by binding enhancer box sequences (E-boxes) [27]. Moreover, c-Myc can lead to genomic instability, gene amplification, cellular proliferation, and repression of apoptosis, which is observed in various tumors, including breast, lung, colon, and prostate cancers [25, 27]. Although numerous studies have examined the interaction of PVT1 and MYC, the detailed mechanisms of the interaction between them remain unclear. In this review, we update the more recent findings for PVT1 and MYC, with the aim of promoting modern studies and the development of clinical therapies.

## Cancer risk related to *PVT1* fusion gene on 8q24

**Fig. 1:**
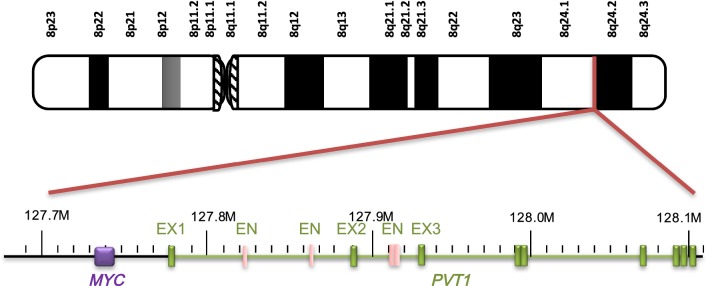
Location of *PVT1* and *MYC* on 8q24. *EX* exon, *EN* enhancer

Both human *PVT1* and *MYC* are located at the 8q24 locus, a well-known cancer-associated chromosome segment and a common and preferred integration site for somatic cell expansion in many cancers (Fig. 1), such as prostate, colorectal, breast, ovarian, and cervical cancers [28–32]. The fragile sites are heritably specific chromosomal loci. When cells are undergoing mitosis, the chromatin region exhibits a loose state and high DNA flexibility, and the induction of fragile sites inhibits partial replication without blocking the cell cycle [33]. There are two common fragile sites in the 8q24 segment, FRA8C and FRC8D, where substantial amounts of DNA helix flexibility exist. When cells are under the influence of genetic changes or exposed to factors that interfere with DNA replication, such as hypoxia, viral invasion, and cytotoxic drugs, these sites induce chromosome breakage and amplification, translocation, and other chromosome structural variations [34]. As a gene sequence adjacent to the fragile site, *PVT1* is also prone to gene rearrangement within the chromosome or interchromosomal segments during gene amplification, leading to an increase in its copy number. Cytogenetic analysis indicated that gene amplification was driven by recurrent breakage within the common fragile site via a break–fusion–bridge mechanism [35, 36]. Although no studies have specifically reported the mechanism of the increased *PVT1* copy number, we predict that its amplification occurs through this general amplification mechanism at the fragile sites.

Amplification of the *PVT1* locus often manifests as the fusion of sister chromatid ends to form double minute chromosomes or a homogeneously staining region [36]. Through either amplification process, *PVT1* will generate new fusion genes, and these abnormal changes in the gene replication process play an important role in tumor development [37]. Numerous studies have shown that *PVT1* can fuse to other genes by translocation in certain tumor tissues (Table 1). Although *PVT1* is a nonprotein encoding gene, the fusion of other genes may produce abnormal protein that participates in the cancer process. For example, in small cell lung cancer, exon 1 or 2 of *PVT1* fuses to exon 2 of *AKT3* (1q44) to form a chimeric transcript *PVT1*-*AKT3* [38]. It encodes a shorter AKT3 protein with a missing N-terminus and an incomplete PH domain, which changes the function of the protein and promotes tumor progression [38]. We also found that gene fusion of *PVT1* with its neighboring gene *MYC* is most common in 8q24-amplified cancers [37, 39]. An increased *MYC* copy number and *PVT1* expression occur in more than 98% of cancer cases with increased 8q24 copy numbers, which points to an interaction between *PVT1* and *MYC* and that they are part of a common signaling pathway [40]. False DNA repair, referred to as chromothripsis, may occur during amplification of the *PVT1* locus, producing fragmented 8q24 amplicons. In different tumors, these fragments, including *PVT1* and *MYC*, fuse with different exons [39]. Thus, *MYC* can be regulated by the *PVT1* promoter because of the loss of its partial exons [39]. In mice artificially transfected with copies of *MYC*-*PVT1*, the level of RSPO1, a key regulatory molecule in the Wnt/β-catenin pathway, was upregulated. Accelerating proliferation of breast cancer cells in mice was subsequently observed, and the increased copy number of each single gene separately did not have a proliferative effect [41]. Therefore, most studies have shown that *PVT1* fusion genes exert the effect of affecting cancer risk mainly by driving protein expression. With respect to whether it affects the function of lncRNA, additional research is expected.

**Table 1:** Fusion genes of *PVT1* in malignancy

Cancer type	Fusion locus	Fusion gene	References
Colorectal cancer	8q24/8q23.1	*PVT1*-*RSPO2*	[42]
	8q24/8q24.21	*PVT1*-*MYC*	[37]
Neuroendocrine bladder cancer	8q24/17q12	*PVT1*-*ERBB2*	[43]
Acute myeloid leukemia	8q24/8q24.21	*PVT1*-*CCDC26*	[44]
	8q24/8q24.13	*PVT1*-*NSMCE2*	[45, 46]
Gastric cancer	8q24/11p13	*PVT1*-*PDHX*	[47]
	8q24/10q26.13	*PVT1*-*ATE1*	[47]
	8q24/11p13	*PVT1*-*APIP*	[47]
	8q24/10q26.12	*PVT1*-*PPAPDC1A*	[47]
Blastic plasmacytoid dendritic cell neoplasm	8q24/6p21	*PVT1*-*SUPT3H*	[48]
Burkitt lymphoma	t(2,8)or t(8,22)	*PVT1*-*IGγ or IGk*	[49]
Small-cell lung cancer	8q24/8q13.3	*PVT1*-*EYA1*	[50]
	8q24/8q12	*PVT1*-*CHD7*	[51]
	8q24/1q44	*PVT1*-*AKT3*	[38]
Multiple myeloma	8q24/13q13	*PVT1*-*NBEX*	[38]
	8q24/16q23	*PVT1*-*WWoX*	[38]
Medulloblastoma	8q24/8q24.21	*PVT1*-*MYC*	[39]
	8q24/8q24.3	*PVT1*-*NDRG1*	[39]

From the perspective of genetic epidemiology, genome-wide association studies (GWAS) indicate that 8q24 is a cancer susceptibility locus and single nucleotide polymorphisms (SNPs) in this region are associated with cancers, such as colorectal, prostate, breast, ovarian, and pancreatic cancers [52–58]. The cancer risk-associated SNPs are differentially located on 8q24 in different cancers; for example, rs1561927 in pancreatic cancer locates 455 kb telomeric of *PVT1*, while rs10088218 in ovarian cancer lies 400 kb 3′ of *MYC* [56–59]. Furthermore, it has been shown that the *MYC* allele in cis linked to the cancer risk-associated SNP variant shows significantly higher expression than the *MYC* allele linked to the nonrisk-associated variant [60]. The GWAS study in colorectal cancer samples and normal tissue control samples found that SNP rs6983267 on 8q24 is a transcriptional enhancer [61]. Moreover, the chromosome conformation capture assay demonstrated that the risk region interacted with *MYC* in the long-range physical way, leading to tumor development [61]. At the same time, chromosome conformation capture research also indicated that rs6983267 has interactions with the adjacent *PVT1* promoter [60, 62]. Moreover, rs378854 can also interact with *MYC* or *PVT1* promoter in prostate cancer [63]. The 8q24 region contains multiple independent risk regions of prostate cancer, colon cancer, breast cancer, and pancreatic cancer [64, 65], all of which have been shown to affect distant target genes and thereby increase cancer susceptibility. These risk regions exhibited strong interactions with *MYC* in their respective cancer cell lines, whereas no risk SNP transcription associated with chr8 transcription was found in normal tissues. These observations provide genetic statistical evidence that *PVT1* and *MYC* are relevant to cancer risk at the chromosome level.

## Positive feedback between PVT1 and MYC

Since *PVT1* was discovered and received widespread attention, researchers have closely examined whether it is involved in the cancer-promoting pathway related to *MYC*. A large number of studies showed that PVT1 levels are higher in many cancers with increased MYC expression compared to normal tissues and are associated with poor prognosis [66–68]. Tissue microarray analysis of 8 primary tumors (lung, colon, rectum, stomach, esophagus, liver, kidney, and mammary gland) indicated a high correlation between PVT1 and c-Myc expression, providing strong evidence for the cooperation of PVT1 and MYC in different human cancers [40]. The gene fusion of *MYC* and *PVT1* and the positive feedback mechanism between them can increase the transcription of PVT1, thereby enhancing the role of PVT1 in tumors.

**Fig. 2:**
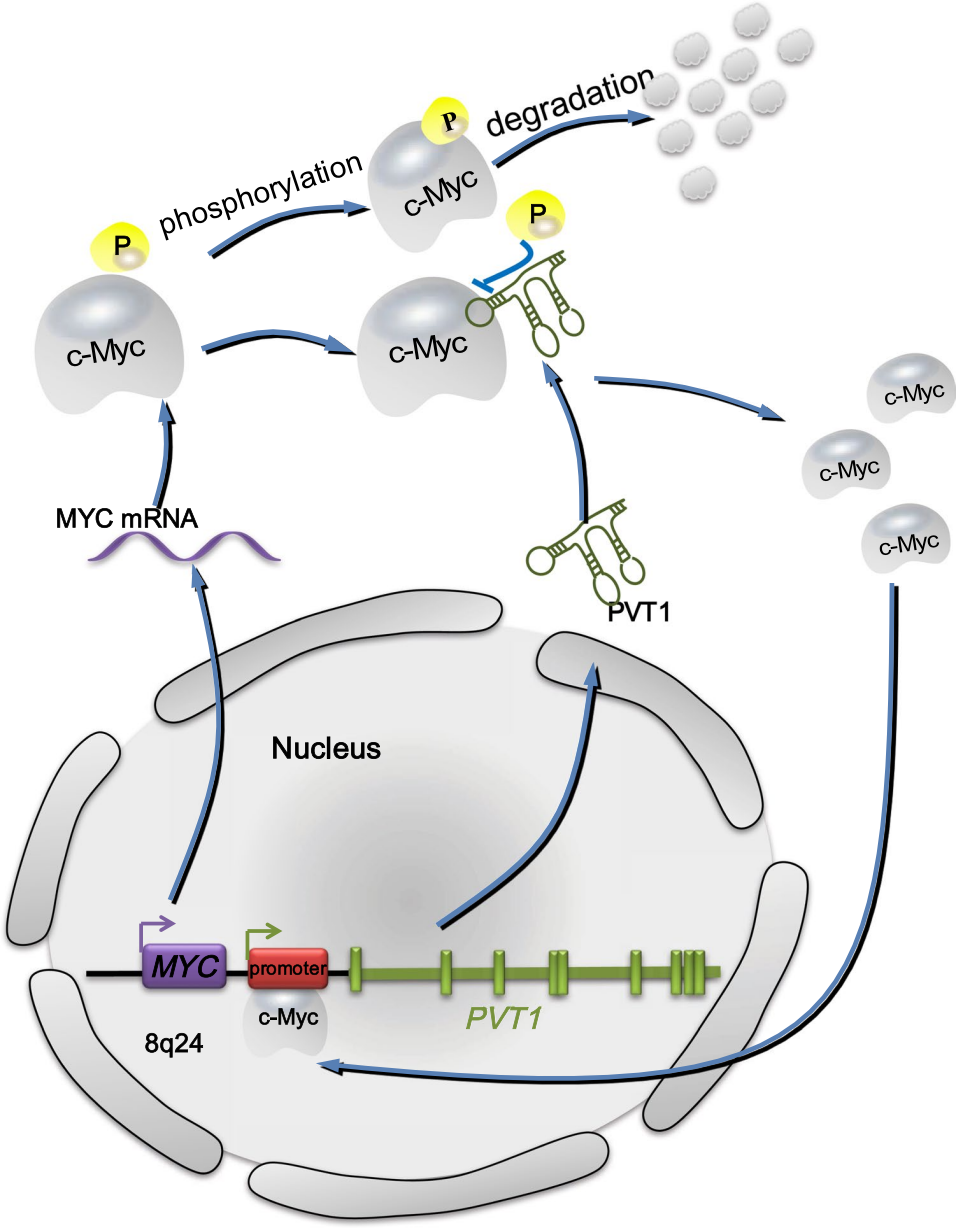
Positive feedback between PVT1 and MYC. C-Myc protein tends to be phosphorylated at the Thr-58 site by GSK3-β and then degraded by proteasome. However, PVT1 promotes c-Myc stability by inhibiting the degradation and phosphorylation of the Thr-58 site of c-Myc. Thus, c-Myc can return to the nucleus to interact with the *PVT1* promoter, leading to the upregulation of PVT1. PVT1, the transcription product of *PVT1*, joins the positive feedback pathway to stabilize c-Myc protein

Interestingly, when siRNA was used to knock down PVT1, low levels of c-Myc were found without significant changes in MYC mRNA levels, which indicates that c-Myc levels are closely related to PVT1 in high-copy 8q24 proliferating cancer cells. Phosphorylation of the threonine 58 (Thr58) site on c-Myc causes the degradation of itself through the ubiquitin proteasome pathway [69]. In tumor tissues, the major transcription product of the *PVT1* gene, PVT1 (an lncRNA), increases the level of c-Myc by enhancing the stability of it via blocking the phosphorylation of the Thr58 site on c-Myc [8, 70, 71]. Furthermore, c-Myc is an important transcription factor that enhances PVT1 transcription. Carramusa et al. [72] found that the *PVT1* promoter region contains two enhancer E-boxes that serve as c-Myc binding sites and E-box 2 clearly mediates the binding of c-Myc to the *PVT1* promoter to promote PVT1 expression. Northcott et al. [39] subsequently confirmed that *MYC* positively regulates the expression of *PVT1*-encoded miRNA in medulloblastoma cells and *MYC* can enhance its own expression through the *PVT1* promoter in tumors with *PVT1*-*MYC* gene fusion. Thus, the mechanism of coamplification of *PVT1* and *MYC* in tumor cells may involve a positive feedback pathway in which c-Myc increases the transcription of PVT1 by binding to E-boxes located in the *PVT1* promoter region, which results in the increased expression of PVT1. PVT1 then prevents the degradation of c-Myc by blocking the phosphorylation of Thr58 in c-Myc and maintains a high c-Myc protein level (Fig. 2). Therefore, through this positive feedback mechanism, PVT1 and c-Myc in cancer cells can remain at a high level, which results in the synergistic promotion of tumorigenesis by PVT1 and c-Myc.

## Promoter-enhancer competition between *PVT1* and *MYC*

It has been known for many years that enhancers play an important role in genomic transcription. Their main function is to enhance the transcriptional initiation rate as a sequence element to enhance promoter activity. Initially, the understanding of enhancer–promoter interactions only existed at the linear level. With the development of high-throughput techniques and chromosome topology spatial structure detection techniques, researchers were able to detect the interaction among genomic components in 3D and discover more complex mechanisms [73, 74]. There are two mechanisms for the spatial interaction between the promoter and enhancer. One mechanism is to connect linearly distant loci with proteins, such as CTCF. In the other mechanism, for the topologically associated domain (TAD) scaffold, contact between the promoter and enhancer can coordinate the regulation of gene expression [75, 76]. Promoter-enhancer competition between *PVT1* and *MYC*, described as follows, mainly occurs in the TAD.

**Fig. 3:**
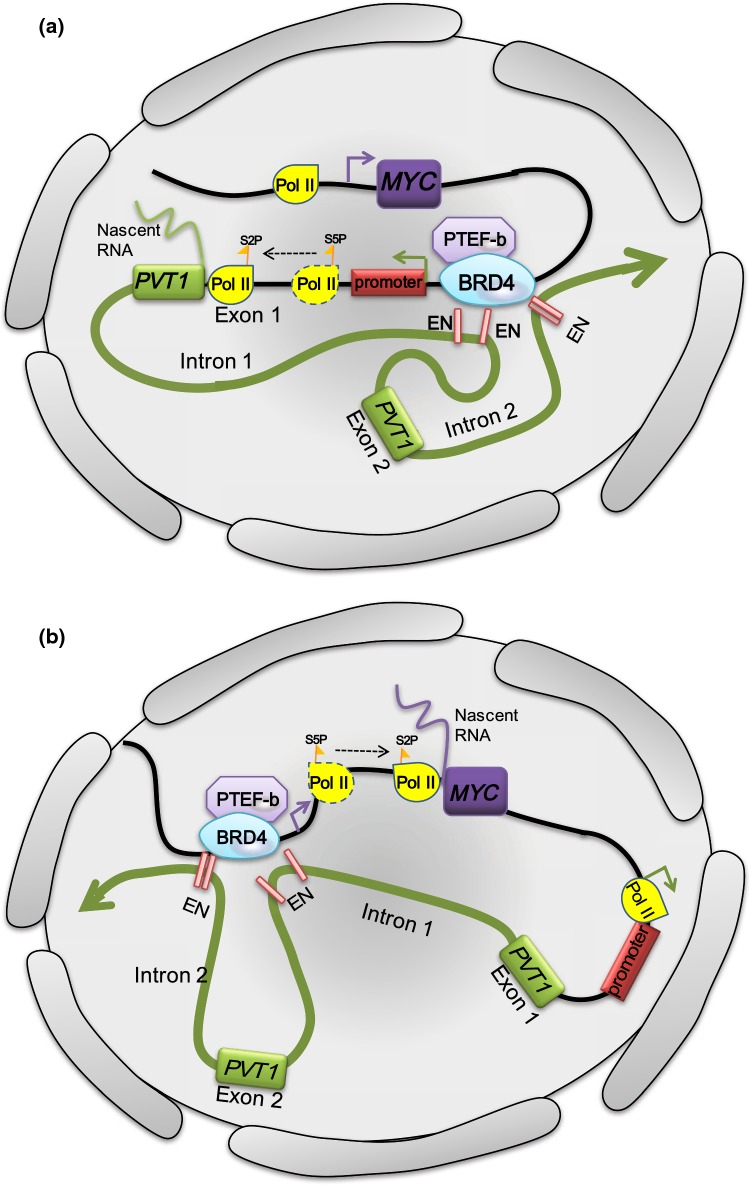
Promoter-enhancer competition between *PVT1* and *MYC.***a** When the *PVT1* promoter functions normally, the internal enhancer of *PVT1* (822E, 866E, 912E, 919E) tends to enhance the transcriptional efficiency of transcription factors starting from the *PVT1* promoter. The transcription depends on phosphorylation of the Ser 2 site of Pol II by BRD4 after phosphorylation of the Ser 5 site and P-TEFb is recruited by BRD4 to restart transcription. **b** In breast cancer and malignant lymphoma cells, the *PVT1* promoter sequence mutates, and the change in the topology enables the *MYC* promoter to interact with enhancer of *PVT1* frequently, upregulating the expression of *MYC* will be

Cho et al. [9] found that the *PVT1* promoter can *cis*-competitively contact its own four intragenic enhancers, reducing competition from the *MYC* promoter and downregulating the expression of *MYC* to inhibit the proliferation of tumor cells. Unexpectedly, they found that when the *PVT1* promoter was silenced, tumor cell proliferation was significantly enhanced, which was contrary to the experimental conclusion from previous studies that “*PVT1* has a cancer-promoting effect” [8, 77]. Moreover, they also found that *MYC* mRNA was significantly upregulated in cells after silencing the *PVT1* promoter and the role of *PVT1* promoter was independent of its lncRNA function, which affects the transcription level of *MYC*. On chromosome 8, the distance between *MYC* and *PVT1* is 53 kb, so how does the *PVT1* promoter affect *MYC* transcription despite this relatively long distance barrier? Cho et al. used the Hi-ChIP method to target the enhancer-associated marker histone 3 lysine 27 acetylation. The promoter of *PVT1* and its four intragenic enhancers and the *MYC* promoter are in the same TAD of discrete self-interacting units of three-dimensionally organized chromatin [78]. Normally, the four intragenic enhancers of *PVT1* contact their own *PVT1* promoter. However, in cells in which the *PVT1* promoter is silent, the enhancers of *PVT1* can contact the *MYC* promoter more readily and reduce their association with the *PVT1* promoter. Silencing the enhancers of *PVT1* could reverse the upregulation of *MYC* induced by silencing of the *PVT1* promoter. According to the previously described experimental results, a “promoter-enhancer competition” model was proposed (Fig. 3), which sheds light on the recurrent chromosomal rearrangements within the *MYC*-*PVT1* locus.

The transcription of *PVT1* and *MYC* starts at the promoter core sequence for both genes, and RNA polymerase II (Pol II) is recruited by the transcription complex and accurately locates the specific region [79]. There is a promoter proximal pause mediated by a transcriptional pause-inducing factor during transcriptional elongation in RNA synthesis, and the epigenetic reading protein bromodomain-containing protein 4 (BRD4) recruits P-TEFb, a kinase-positive transcription elongation factor, to the pretranscriptional initiation complex [80]. BRD4 can bind to the carboxy terminal domain of Pol II and phosphorylate it at the Ser 2 site, and P-TEFb dissociates the pause-inducing factor from the transcription complex to synergistically promote transcription via Pol II [81]. In normal tissues, the four internal enhancers of *PVT1* are preferentially contacted by the *PVT1* promoter; thus, BRD4 will preferentially occupy the *PVT1* promoter, reducing the occupation of BRD4 at the *MYC* promoter and decreasing the transcription of *MYC*. Therefore, the *PVT1* promoter can act as a tumor suppressor DNA element of *MYC*. In human breast cancer and malignant lymphoma, the *PVT1* promoter is mutated. Thus, the *MYC* promoter predominates the promoter–enhancer competition, thereby increasing the *MYC* mRNA level (Fig. 3). According to the description of the topology structure, the *PVT1* promoter, *MYC* promoter, and the four enhancers belong to the same TAD in normal tissues, while in cancer cells, the circumstance is different. Mutations in the *PVT1* promoter lead to changes in chromosome 3D construction so that the four intragenic enhancers of *PVT1* become closer to the *MYC* promoter and preferentially contact it. Thus, the promoter of *PVT1* inhibits the expression of its “neighbor” gene *MYC* by competing for the four enhancers on the same chromosome. We hypothesize that the “promoter–enhancer competition” model is the initial step in the *MYC*-driven carcinogenic process. The high level of *MYC* transcripts is involved in the positive feedback mechanism previously described, activates the *PVT1* promoter and promotes PVT1 expression, which is consistent with the high expression of both MYC and PVT1 in tumor cells.

Additionally, in some tumor tissues, the region near the *PVT1* promoter shows more pronounced structural changes than other lncRNA promoters, such as deletions, inversions, or duplications, which alter the chromatin and TAD environment of the *PVT1* promoter [9]. In human (ER−) HER2+ breast cancer, the *PVT1* intron 1, which is close to the *PVT1* promoter, is abnormally prone to cleavage, which might result in the fusion of *PVT1* with another gene on the same chromosome leading to an abnormal transcription process not regulated by the *PVT1* promoter.

In normal cells, studies have demonstrated low expression of PVT1 and *MYC* [72]. If the “promoter–enhancer competition” between *PVT1* and *MYC* commonly exists in cells, the *PVT1* promoter serves as a tumor suppressor, and will further downregulate the expression of *MYC*, preventing the development of *MYC*-driven tumors. Thus, the mutation of *PVT1* promoter will be the last straw in cancer development. However, the “promoter–enhancer competition” between *PVT1* and *MYC* is currently only observed in breast cancer cell lines and mutation of the *PVT1* promoter is only observed in breast cancer and malignant lymphoma. Thus, whether the competition mechanism acts as a complement and improves the positive feedback mechanism in common *MYC*-driven tumors requires further analysis.

## PVT1 participates in the downstream signaling pathway of c-Myc by regulating key molecules

**Fig. 4:**
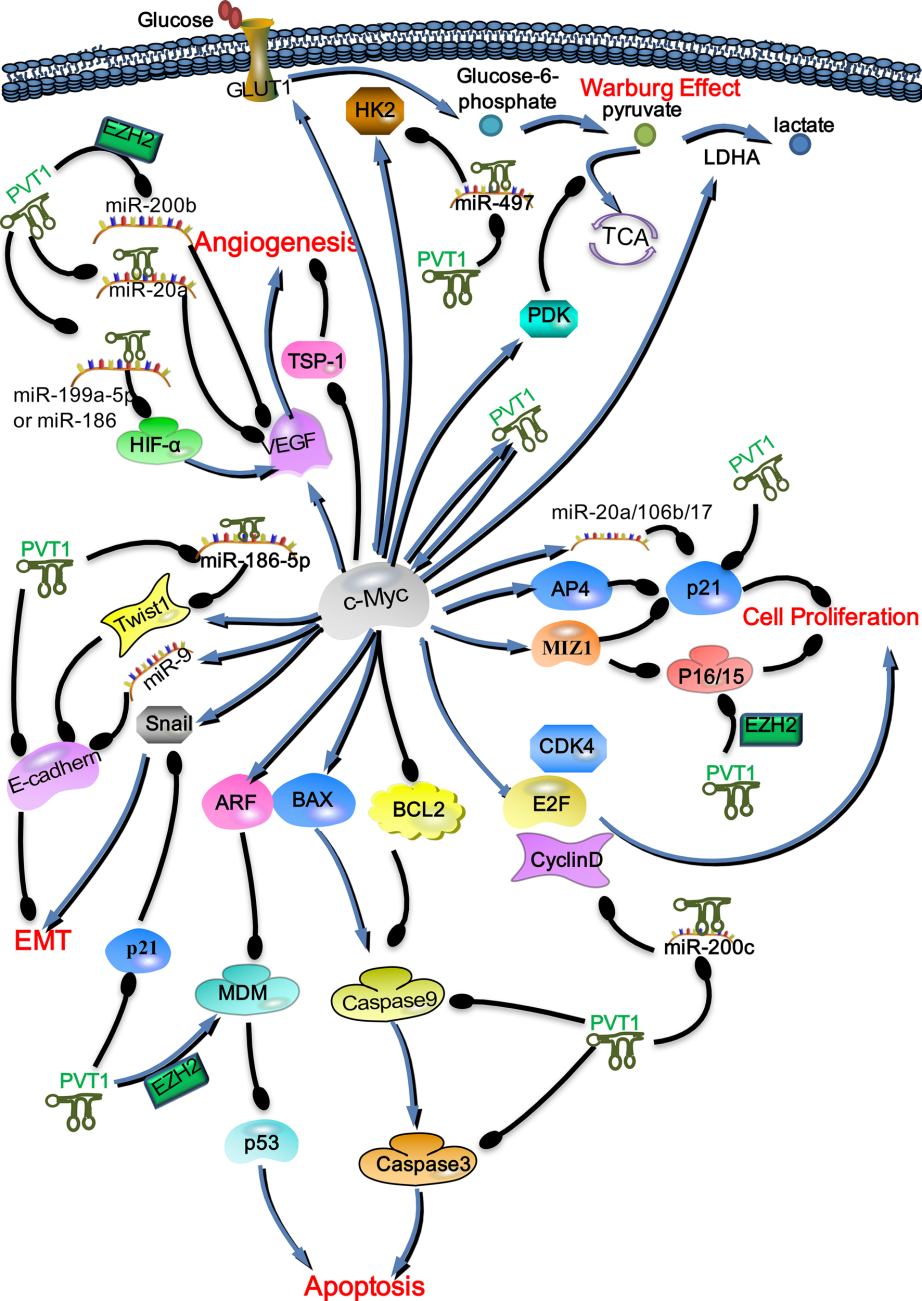
PVT1 participates in the downstream signaling pathway of c-Myc by regulating key molecules. In cancer cells, PVT1 not only exhibits a positive feedback interaction with c-Myc but also participates in the signal pathways downstream of c-Myc to promote the occurrence and development of tumors. PVT1 participates in the downstream signaling pathway of c-Myc by regulating the expression of key molecules. PVT1, similar to c-Myc, can promote tumor proliferation, angiogenesis, the EMT and the Warburg effect. Moreover, PVT1 may inhibit c-Myc-induced apoptosis by regulating caspase9, caspase3, and MDM. In this figure; the blue arrow indicates promotion/upregulation, while the black line indicates inhibition/downregulation

In addition to preventing the phosphorylation of c-Myc to maintain its stability, PVT1 can participate in the downstream signaling pathway of c-Myc by regulating its downstream key factors (Fig. 4). PVT1 may promote the tumorigenesis and development of tumors together with c-Myc by regulating key molecules in the downstream signaling pathway of c-Myc. Therefore, we adequately understand that PVT1 and c-Myc jointly regulate key molecules, which may contribute to the development of new and selective anti-cancer drugs for the targeted therapy of cancers with high expression of PVT1 and c-Myc.

### PVT1 participates in the proliferation-related signaling pathway downstream of c-Myc

Malignant cell proliferation is one of the hallmarks of tumor cells [82–86]. Research has shown that c-Myc can promote the transcription of positive cell cycle regulatory factors, such as E2F transcription factors, cyclin D1, and cyclin-dependent kinase 4 (CDK4). They can promote activation of the cell cycle and thus cell proliferation [87–90]. By inhibiting cell cycle inhibitors, such as p15, p16, and p21, which inhibit the cyclin-cdk complex, c-Myc promotes cell proliferation. In cancer cells, a complex of c-Myc and c-Myc-interacting zinc finger protein-1 (Miz1) act on the promoter of p15 and p16, which, in turn, downregulates the expression of p15 and p16, enabling the cell to enter the cell cycle phases. By binding to Miz1, c-Myc can also inhibit the expression of p21 [91]. C-Myc also inhibits the expression of p21 through miR-17, miR-20a, and miR-10b [92, 93]. Moreover, the transcription factor AP4 (TFAP4), induced by c-Myc, binds to the promoter of p21 and inhibits the expression of p21 [94, 95].

Recent studies showed that PVT1 affects the c-Myc-related pathway and influences the cell cycle. PVT1 can promote tumor proliferation by downregulating the expression of p15 and p21 [96]. PVT1 acts on the promoter of *P15* and *P16* by binding the enhancer of zeste homolog 2 (EZH2) to inhibit p15 and p16 expression [97]. In cancer cells, knockdown of PVT1 expression inhibits the expression of cyclin D1 and CDK4, which suggests that PVT1 positively regulates the expression of cyclin D1 and CDK4 [98]. PVT1 inhibits the promoter of miR-200c by recruiting EZH2, which leads to an upregulation of cyclin D1 expression [99]. Furthermore, Chen et al. [22] confirmed that circPVT1 competently binds to the miR-125 family and enhances the expression of the transcription factor E2Fs. Therefore, PVT1 participates in the proliferation-related signaling pathway downstream of c-Myc by regulating p21, p15/p16, CD4, and cyclin D (Fig. 4).

### PVT1 participates in the downstream angiogenesis-related signaling pathway of c-Myc

Sustained angiogenesis is a key element in tumorigenesis. In an anoxic environment inside a tumor mass, hypoxia-inducible factor-1 (HIF-1) induces the expression of genes involved in angiogenesis [100–102]. HIF-1α is a crucial oxygen sensor and plays a leading role in angiogenesis. By binding to the promoter of vascular endothelial growth factor (*VEGF*), HIF-1α upregulates the expression of VEGF [103]. VEGF can act on the vascular endothelium, accelerating its proliferation and tube formation and increasing vessel permeability. In the angiogenesis signaling pathway, VEGF-A and thrombospondin-1 (TSP1) are angiogenesis inducers and inhibitors, respectively. TSP1 is a key balance factor in angiogenesis. It also binds to transmembrane receptors on endothelial cells, triggering inhibitory signals that counteract angiogenesis stimulation [104]. In cancer cells, c-Myc increases the transcriptional level of HIF-1α [105], thus increasing the expression of HIF-1α, further promoting the expression of VEGF, which participates in downstream signaling pathways and promotes tumor angiogenesis. c-Myc can also directly upregulate the expression of VEGF [106] and inhibit the transcription of TSP1 [107], further promoting tumor angiogenesis. High expression of PVT1 in tumor cells can be used as a competing endogenous RNA sponge to adsorb miR-199a-5p or miR-186 and subsequently upregulate the expression of HIF-1α [108, 109]. PVT1 can recruit EZH2 to the *miR*-*200b* promoter and inhibit miR-200b expression [110]. MiR-200b reduces the VEGF level [111]. Thus, PVT1 may upregulate the expression level of VEGF through inhibiting miR-200b. Moreover, PVT1 may increase the expression of VEGF by inhibiting miR-20a [13, 112]. Therefore, PVT1 participates in the downstream angiogenesis-related signaling pathway of c-Myc and can promote tumor angiogenesis (Fig. 4).

### PVT1 participates in the downstream epithelial–mesenchymal transition-related signaling pathway of c-Myc

Metastasis of tumors typically leads to patient death [113–117]. Studies have shown that the high expression and amplification of the *MYC* gene can promote tumor metastasis, which is closely related to the epithelial–mesenchymal transition (EMT) of tumor cells [118, 119]. The mesenchymal state is characterized by high expression of vimentin and low expression of E-cadherin. Decreased levels of E-cadherin can result in reduced adhesion of cells, which enables them to acquire characteristics that make them prone to invasion and metastasis. The loss of E-cadherin expression and upregulation of snail homolog 1 (Snail1), Twist1, and a number of other proteins are considered important features of the EMT. In tumor cells, the TGF-β signaling pathway induces Snail, which further inhibits the expression of E-cadherin, resulting in a loss of adhesion between cells. After knocking out *MYC*, the expression of Snail induced by the TGF-β signaling pathway decreased because c-Myc promoted the transcription of Snail and stabilized it [119, 120]. Studies have shown that c-Myc can induce the production of miR-9, which promotes the migration and invasion of cancer cells by inhibiting the cell adhesion protein E-cadherin [118, 121, 122]. Twist1 has been shown to promote the EMT by directly inhibiting epithelial markers, such as E-cadherin, and upregulating mesenchymal markers, such as N-cadherin [123]. Twist1 is the downstream transcription target of c-Myc, and c-Myc can directly enhance the transcription of Twist1 [124].

Studies have shown that elevated expression of PVT1 can induce a decrease in E-cadherin [125, 126]. Inhibition of the expression of PVT1 in cancer cells reduces the expression of vimentin, while it enhances the expression of E-cadherin [98]. PVT1 downregulates the expression of p21, while p21 downregulates the expression of Snail [127]. Moreover, PVT1 can upregulate Twist1 by adsorption of miR-186-5p to promote the EMT [128]. Therefore, PVT1 participates in the proliferation-related signaling pathway downstream of c-Myc by affecting Snail1, Twist1, and E-cadherin (Fig. 4).

### PVT1 participates in the downstream Warburg effect-related signaling pathway of c-Myc

Cancer is often accompanied by deregulating cellular energetic [129–133]. The pyruvate produced by glycolysis does not enter the mitochondria and is converted to lactic acid by lactate dehydrogenase (LDH), which typically occurs when normal cells are in a low-oxygen state. However, in cancer cells, the production of lactic acid under aerobic conditions is referred to as “aerobic glycolysis” or the Warburg effect. Many genes involved in glucose metabolism have been reported to be directly upregulated by the induction of c-Myc, particularly glucose transporter 1 (GLUT1) and hexokinase 2 (HK2) [134]. C-Myc also regulates pyruvate dehydrogenase kinase (PDK), which blocks pyruvate from entering the TCA cycle. C-Myc activates lactate dehydrogenase A, which converts pyruvate to lactic acid. PVT1 acts as an endogenous competitive RNA to inhibit miR-497 whose downstream target is HK2, which results in elevated HK2 expression, increasing glucose consumption and glycolysis [135]. Therefore, PVT1 can regulate HK2 expression to participate in the downstream Warburg effect-related signaling pathway of c-Myc (Fig. 4).

### PVT1 participates in the downstream apoptosis-related signaling pathway of c-Myc

In most human cancers, c-Myc expression is deregulated and/or significantly increased. Interestingly, high levels of c-Myc overexpression not only induce ARF transcription but also stabilize ARF by inhibiting ubiquitin ligase, which, in turn, leads to p53 activation, induces apoptosis, and limits c-Myc-induced carcinogenesis [136]. Mouse double minute 2 homolog (MDM2) downregulates p53 by binding to and inhibiting the transactivation domain of p53 and promoting its degradation [137]. However, ARF can promote the degradation of MDM2, which, in turn, leads to p53 activation. Inactivation of the Arf-Mdm2-p53 tumor suppressor pathway is believed to be a crucial step in tumorigenesis [138]. C-Myc induces apoptosis by regulating the ARF-MDM2-p53 pathway to limit its own carcinogenic potential [136]. However, PVT1 can increase the stability of EZH2 protein by binding to EZH2, which promotes the expression of MDM2 protein. In cancer cells, high expression of PVT1 enhances the expression of EZH2 and MDM2, inhibits the expression of P53 protein in cancer cells, and thus exerts anti-apoptotic effects [139, 140]. C-Myc can also inhibit anti-apoptotic proteins, such as BCL-2 and BCL-XL, leading to a certain amount of tumor apoptosis [141]. When c-Myc exerts a pro-apoptotic function, it can further activate caspase-9 and caspase-3 to induce apoptosis by increasing the expression of and activating the pro-apoptotic protein Bax [142]. PVT1 can downregulate the expression of caspase-9, caspase-7, and poly ADP-ribose polymerase, thereby inhibiting apoptosis and ultimately leading to radiation tolerance [14]. Studies have also shown that PVT1 can downregulate the expression of caspase-3 to inhibit apoptosis [143, 144]. Therefore, PVT1 may participates in the downstream apoptosis-related signaling pathway of c-Myc by affecting MDM, caspase-9, and caspase-3 (Fig. 4).

## Perspectives

In addition to the interaction between PVT1 and MYC, the newly discovered molecule CircPVT1 is also associated with MYC [145–149]. CircPVT1 is highly expressed in cancer and can mediate the expression of c-Myc [22, 150]. Let-7 can target *MYC* mRNA and downregulate its expression; however, circPVT1 can impede let-7 through its sponge adsorption effect and increase c-Myc expression [22, 151]. In acute lymphocytic leukemia, circPVT1 can upregulate the expression of c-Myc and anti-apoptotic Bcl-2 proteins [23]. After deletion of circPVT1, the expression levels of c-Myc and Bcl-2 proteins were significantly reduced, while the level of PVT1 RNA was not changed [23]. However, CircPVT1 has many other mechanisms that have not been elucidated. It is worth further investigating whether CircPVT1 has similar mechanisms to PVT1 in cancer cells, whether CircPVT1 interacts with MYC, and which molecules CircPVT1 can regulate to participate in the downstream signaling pathway of c-Myc. Moreover, there are no studies on PVT1-encoded microRNAs that indicate whether they interact with MYC (including miR-1204, miR-1205, miR-1206, miR-1207-5p, miR-1207-3p, and miR-1208) [20, 21].

Currently, the mechanism of action of PVT1 in cancer and the interaction mechanism with MYC are not completely clear; however, in-depth studies of PVT1 can contribute to the development of new therapeutic targets. C-Myc is an important protein in cells and participates in many important metabolic pathways. Overexpression of c-Myc in cancer substantially enhances certain metabolic pathways. If c-Myc is directly inhibited, therapeutic interventions will have a strong impact on patients [152]. Therefore, utilizing a small molecular drug to inhibit PVT1, compared to treatment that directly targets MYC, would fine-tune the level of c-Myc in cancer and reduce toxic side effects or exert an influence on the c-Myc downstream biological phenotype, which is another therapeutic target in *MYC*-driven cancer. Recently, antisense LNA GapmeRs have been used by researchers to degrade PVT1 in acute erythroleukemia cell lines, which can increase apoptosis and necrosis of tumor cells. They suggested that PVT1 antisense LNA GapmeRs can be used alone or combined with chemotherapeutic drugs in the treatment of acute erythroleukemia [153]. PVT1 promotes the development of cisplatin resistance in colorectal cancer; thus, silencing PVT1 inhibits tumorigenesis and cisplatin resistance in colorectal cancer [154]. Knockdown of PVT1 to a certain extent enhances the radiosensitivity of non-small cell lung cancer cells through inhibiting cell proliferation and promoting apoptosis, which provides a new therapeutic target for improving the efficiency of radiotherapy in patients with non-small cell lung cancer [155]. In addition, studies have found that gemcitabine can inhibit the growth of pancreatic cancer cells by decreasing PVT1 levels and increasing *PVT1* encoded miRNAs, such as the miR-1207 pair (miR-1207-5p/3p) [156, 157]. Therefore, there is a bright future for research on how to use PVT1 as a therapeutic target. It is necessary to develop drugs that target PVT1. In the future, we can interfere with the function of PVT1 in tumor cells using siRNAs or antisense LNA GapmeRs against PVT1. Further in vivo studies and clinical trials are required to evaluate the feasibility of this strategy. Moreover, according to the latest promoter competition mechanism, the *PVT1* promoter, as a “non-standard” tumor suppressor, is a novel candidate for designing a new therapeutic strategy for tumors in which *MYC* serves as a carcinoma driver.

Furthermore, the high and specific expression of PVT1 in human cancer indicates its potential as a biomarker in early clinical diagnosis. PVT1 is considered an indicator of poor prognosis in cancer because PVT1 has been shown to play a role in cell proliferation, cell apoptosis, and cell migration, which are key elements of poor prognosis in human cancer [13, 128]. The expression of PVT1 is upregulated in gastric cancer tissues and significantly associated with advanced tumor and lymph node metastasis. Upregulated PVT1 in gastric cancer can promote the proliferation and invasion of gastric cancer cells, which is associated with poor prognosis [97, 108, 158]. A high PVT1 expression level in patients with prostate cancer has been shown to be associated with low overall survival. PVT1 expression is significantly correlated with tumor stage and can promote tumor cell proliferation, invasion, and metastasis in prostate cancer [128, 144]. The expression of PVT1 in ovarian cancer tissues is higher than that in normal ovarian tissue and is related to the advanced stage of ovarian cancer and lower overall survival. The high expression of PVT1 in ovarian cancer cells promotes the proliferation, migration, and invasion ability of ovarian cancer cells [159, 160]. In gliomas, PVT1 is also highly expressed. PVT1 can promote the development of glioma cells through various mechanisms, resulting in a worse prognosis for patients with glioma and high PVT1 expression [18, 161, 162]. Multiple meta-analyses have also shown that PVT1 can be used as a new tumor biomarker and a predictor of poor prognosis in different cancers [163–167].

## Conclusions

We have summarized the latest research regarding the relationship between PVT1 and MYC. At the chromosomal level, *PVT1* is easily fused with other genes to form a fusion gene that drives the abnormal expression of cancer-associated proteins. Moreover, GWAS also indicated that *PVT1* and *MYC* located in the 8q24 segment are associated with cancer risk. Furthermore, there is a synergistic effect of positive feedback between MYC and PVT1, which results in increased expression levels of these genes in cancer. With the development of 3D technology, the latest research shows that *PVT1* and *MYC* have a “promoter-enhancer competition mechanism” in a three-dimensional structure. Moreover, PVT1 can also participate in the c-Myc downstream signaling pathway by acting on key molecules downstream of c-Myc.

However, the interaction between PVT1 and MYC has not been fully understood, and further studies are needed. Studies have shown that PVT1 can inhibit the phosphorylation and degradation of c-Myc to promote its stability; however, are there ways that PVT1 impacts the transcription and translation of c-Myc? In addition to gene fusion, the positive feedback mechanism, coamplification and the “promoter-enhancer competition mechanism”, are there other mechanisms of that mediate the PVT1 and MYC interaction? The “promoter-enhancer competition” between *PVT1* and *MYC* is currently only found in breast cancer cell lines and, thus, requires further exploration regarding whether this competition commonly exists in other *MYC*-driven cancers. Moreover, the key molecules that are at the c-Myc downstream signaling pathway and regulated by PVT1 are needed to further verify and clarify to develop anticancer drugs by inhibiting these key molecules. In addition, it is worth elaborating on whether microRNAs and the circPVT1 encoded by *PVT1* interact with MYC.

Although the increasing number of mechanisms of action for PVT1 and its interaction with MYC in cancer are being discovered, there is no application for PVT1 in clinical treatment to date, and only one clinical trial on PVT1 is recruiting subjects. Thus, the findings resulting from such studies are expected to be transformed into methods of early cancer diagnosis and treatment applications in the future.

## Abbreviations

PVT1: Plasmacytoma variant translocation 1
MYC: *MYC* or c-Myc
lncRNA: Long non-coding RNA
E-boxes: Enhancer box sequences
GWAS: Genome-wide association studies
SNPs: Single nucleotide polymorphisms
TAD: Topologically associated domain
BRD4: Bromodomain-containing protein 4
CDK4: Cyclin-dependent kinases 4
Miz1: Myc-interacting zinc finger protein-1
Snail1: Snail homolog 1
TFAP4: Transcription factor AP4
EZH2: Enhancer of zeste homolog 2
TSP1: Thrombospondin-1
VEGF: Vascular endothelial growth Factor
HIF-1: Hypoxia inducible factor-1
LDH: Lactate dehydrogenase
GLUT1: Glucose transporter 1
HK2: Hexokinase 2
PDK: Pyruvate dehydrogenase kinase
MDM2: Mouse double minute 2
